# Expanding Video Consultation Services at Pace and Scale in Scotland During the COVID-19 Pandemic: National Mixed Methods Case Study

**DOI:** 10.2196/31374

**Published:** 2021-10-07

**Authors:** Joseph Wherton, Trisha Greenhalgh, Sara E Shaw

**Affiliations:** 1 Nuffield Department of Primary Care Health Sciences University of Oxford Oxford United Kingdom

**Keywords:** technology-enabled care, video consultations, quality improvement, COVID-19, PERCS framework

## Abstract

**Background:**

Scotland—a country of 5.5 million people—has a rugged geography with many outlying islands, creating access challenges for many citizens. The government has long sought to mitigate these through a range of measures including an ambitious technology-enabled care program. A strategy to develop a nationwide video consultation service began in 2017. Our mixed methods evaluation was commissioned in mid-2019 and extended to cover the pandemic response in 2020.

**Objective:**

To draw lessons from a national evaluation of the introduction, spread, and scale-up of Scotland’s video consultation services both before and during the pandemic.

**Methods:**

Data sources comprised 223 interviews (with patients, staff, technology providers, and policymakers), 60 hours of ethnographic observation (including in-person visits to remote settings), patient and staff satisfaction surveys (n=20,349), professional and public engagement questionnaires (n=5400), uptake statistics, and local and national documents. Fieldwork during the pandemic was of necessity conducted remotely. Data were analyzed thematically and theorized using the Planning and Evaluating Remote Consultation Services (PERCS) framework which considers multiple influences interacting dynamically and unfolding over time.

**Results:**

By the time the pandemic hit, there had been considerable investment in material and technological infrastructure, staff training, and professional and public engagement. Scotland was thus uniquely well placed to expand its video consultation services at pace and scale. Within 4 months (March-June 2020), the number of video consultations increased from about 330 to 17,000 per week nationally. While not everything went smoothly, video was used for a much wider range of clinical problems, vastly extending the prepandemic focus on outpatient monitoring of chronic stable conditions. The technology was generally considered dependable and easy to use. In most cases (14,677/18,817, 78%), patients reported no technical problems during their postconsultation survey. Health care organizations’ general innovativeness and digital maturity had a strong bearing on their ability to introduce, routinize, and expand video consultation services.

**Conclusions:**

The national-level groundwork before the pandemic allowed many services to rapidly extend the use of video consultations during the pandemic, supported by a strong strategic vision, a well-resourced quality improvement model, dependable technology, and multiple opportunities for staff to try out the video option. Scotland provides an important national case study from which other countries may learn.

## Introduction

### Background

The first documented video-mediated medical consultations in health care were conducted in the 1950s via closed-circuit television [1]. While telephone consultations have long been offered in both primary and secondary care, until the COVID-19 pandemic only a tiny fraction of clinicians had ever conducted a consultation by video and an even smaller fraction of patients had received care this way [2-7]. The video consultation is thus of academic interest as an example of a promising service-level innovation that has taken decades to catch on. We initially flagged the pandemic as an “opportunity in a crisis” for giving video consulting the push it needed [8]. In this paper, we consider how one country’s efforts fared.

Prepandemic research on the acceptability, effectiveness, and cost-effectiveness of video consultations seemed to convey a positive message but was potentially misleading. Numerous research trials (generally small in size, parochial in setting, and led by a local enthusiast), in which a digitally confident and low-risk sample of patients selected from a much more diverse clinic population was randomized to continuing their usual outpatient care or trying the video option, usually showed that the latter group did no worse clinically and were no less satisfied than the former, and that costs (when measured) were similar [9-14]. Almost all such studies were underpowered to test their central hypothesis.

In published research trials of video consultations, the service was usually available only as part of the trial and discontinued thereafter, so the challenges of embedding it in business-as-usual were never addressed. Any conclusion that video is effective, acceptable, and safe was therefore naïve and premature. To “work” in the real world, video consultations require new infrastructure, new technologies, new clinical and support roles, new organizational routines, new approaches to privacy and information governance, new clinical techniques (eg, for remote examinations and safeguarding conversations), and new payment and reimbursement frameworks—not to mention measures to improve the digital skills of staff and patients and mitigate digital exclusion [15-20]. Given these complexities, it is small wonder that efforts to rollout video consultation services even following a successful trial or pilot study progressed slowly or not at all [18,21].

Early in the pandemic, an overview pointed out that despite the technical potential of remote forms of consulting to help address infection control,“[m]ost countries … lack a regulatory framework to authorize, integrate, and reimburse telemedicine services, including in emergency and outbreak situations” [22] (p1). But as it turned out, the global emergency gave governments reason enough to cut red tape—that is, remove regulatory blocks to rapid purchase and use of newly developed technologies, especially bespoke software for supporting video consultations [23]. During the first wave of the pandemic, most countries saw a rapid reduction in face-to-face medical consultations and an increase in remote ones in both primary and secondary care [3,24]. Such shifts were part of a system-wide response in what has been termed the world’s first “digital pandemic,” which included technologies to support outbreak monitoring and management, triaging and severity assessment, ordering and documentation, secure messaging, real-time data analytics, fast-track research trials, global knowledge sharing, and living systematic reviews [25-27]. But as Gkeredakis et al [28] (p2) have observed, while the pandemic provided fertile soil for rapid growth of new technologies, “the shifts in digital technology use to cope with the COVID-19 crisis are fast-paced, dramatic and not well understood,” and successful embedding and use of novel solutions is “contingent upon the openness, distributedness, recombinability, re-programmability, and accessibility of digital technologies”.

In these early months of the pandemic, politicians, policymakers, and the lay press across different countries and regions all emphasized the role of new modalities such as video and e-consultations in this unprecedented service change [29-33]. But despite this focus on the novel, the reality in most countries was that most remote consultations—especially in primary care—occurred using the old-fashioned telephone [34-38].

An important question for researchers is why, even in the context of an unprecedented global emergency, establishing and sustaining video consultations as business-as-usual in a mainstream health service has proved such a stubborn challenge. Rather than analyze failures, we have chosen to consider the case of Scotland, UK, as—broadly speaking—a success both before the pandemic (when steady incremental progress was being made to introduce the video option region by region and service by service) and during it (when a rapid and dramatic increase in video consultations was achieved at pace and scale).

In telling Scotland’s story, we draw inspiration from a theoretical approach called appreciative inquiry, a form of action research or action evaluation which explicitly seeks to highlight, learn from, and reinforce the positive—things that went right, preconditions that helped, people who made a difference, and so on—while also identifying and learning from less successful aspects of the case [39]. Appreciative inquiry involves building collaborative researcher–practitioner relationships, systematically identifying “the best of what is,” using creativity and experimentation to try to improve things further, and seeking to extend and replicate positive mechanisms and outcomes across the system.

The aim of this study was to draw lessons from an in-depth study of one country that could inform video consultation services and policy decisions more widely. In the remainder of this paper, we first give a historical background to the Scottish case. In the “Methods” section, we describe our aims, study setup, research questions, theoretical framework (Planning and Evaluating Remote Consultation Services [PERCS]), data sources, and methodological approach for our national evaluation of video consultation services both pre- and peripandemic. We then describe our findings, structured along the 7 domains of the PERCS framework. Finally, we discuss the Scottish case in the context of the wider literature, highlighting learning points for other countries.

### Scotland: A National Case Study of Technology-Enabled Care

Scotland (population 5.5 million) is 1 of the 4 jurisdictions in the United Kingdom with a land area only 40% smaller than England (population 56 million). Much of it is mountainous and rugged with lakes (known as lochs), rivers, and offshore islands. Scotland thus shares some of the geographical challenges of remote Scandinavian regions, with some journeys involving a combination of land, water, and air. Scotland’s health service is organized separately from those of England, Wales, and Northern Ireland. Health and care services are mainly delivered by 14 territorial health boards and are underpinned by a strong public-sector ethos which emphasizes professionally led quality improvement and reducing inequalities [40]. Scotland resisted the purchaser–provider split, which has existed in England and Wales since 1991 and created additional hurdles for introducing new technologies [41].

There has long been a strategic intent in Scotland to support and extend remote consulting options, including via video. This was advocated from 2008 through Scotland’s eHealth Strategy [42,43] and extended in a more recent Digital Health and Care Strategy [44]. In all these documents, remote care is framed as a means to improve citizens’ access to services and, ultimately, to improve outcomes and potentially reduce inequalities. In 2014, the Scottish Government established the Technology-Enabled Care (TEC) Programme to drive the widespread adoption of technology to support self-management of illness (eg, self-monitoring of long-term conditions) as well as improve access to professional care, partly in response to the perceived need for service transformation in the context of rising demand for both health and social care. The TEC Programme aims to support local deployment as well as strengthening national technical and support infrastructure.

The video consulting workstream of the TEC Programme was seen as enabling pooling of expertise and provision across the country to ensure a high-quality patient experience. Initially, this involved various pilot studies which used different video technologies, including Cisco Jabber and Polycom devices, before the TEC team decided in 2015 to introduce a more bespoke product (Attend Anywhere), described below. Based on the success of a pilot co-design and quality improvement program in one health board (Highland) in 2017, the video consulting service using the Attend Anywhere platform was branded nationally as “Near Me” (a name chosen by a patient). In November 2018 the TEC Programme launched a £1.6 million (US $2.3 million) “scale-up challenge,” to support wider rollout across all health boards. By February 2020, all 14 health boards and the Golden Jubilee National Hospital (the main tertiary referral center based in Glasgow) were enrolled in the program.

Even before the pandemic, the Near Me video service had been adopted by about 180 services, spanning 35 different clinical specialties, albeit at different levels of implementation. But actual use of video within most of these services remained relatively low, with many clinicians describing their use of it as “ad hoc” rather than business-as-usual. Nevertheless, the scale-up effort continued to progress and established a strong national profile, steadily working through regulatory, infrastructural, and operational challenges. The rationale for scaling up Near Me was initially reducing patient travel and improving access and service efficiency [45]. Video consulting was generally—but not universally—seen as enhancing the existing face-to-face services, rather than replacing them. But in the context of the pandemic, emphasis shifted to infection control and the maintenance of core services.

In March 2020, when the COVID-19 outbreak reached Scotland, most routine and nonurgent care in both primary and secondary care was halted. Space and staffing were repurposed to support the pandemic response. Rollout of the Near Me video service was accelerated via a 12-week scale-up plan, led by a rapidly assembled national implementation team within the existing TEC Programme. Staff were drafted in from across Healthcare Improvement Scotland (a Special NHS Board in Scotland with a remit to help implement health care priorities), the Scottish Access Collaborative (a government program to sustainably improve waiting times for non-emergency procedures), and the Care Inspectorate (a regulatory body for social work and social care services in Scotland). They prepared guidance and resources for deployment of video consultations across a range of health and care settings and built links with other key government departments—for example, with the Primary Care Division which covered general practice. National-level groundwork and strategic planning over the previous 2 years to create technical infrastructure, service readiness, and positive attitudes helped services transform, at pace and scale, to a remote-first mode of operating as the pandemic took hold.

Following this 12-week scale-up, an engagement exercise was undertaken with various service teams to consolidate implementation plans for the video service going forward. A key element of this was the need to streamline and coordinate activities that had been undertaken in the immediate response, as well as leverage learning and resources developed. More broadly, health boards were required to produce recovery plans for clinical services during and beyond the pandemic, as part of the Scottish Government Re-mobilise, Recover, Re-design framework [46].

Evaluation and system learning were built into the Scottish Government’s strategy both before and during the pandemic. Below, we describe the methods and findings of a commissioned evaluation.

## Methods

### Aim and Set Up of the Study

This commissioned evaluation aimed to highlight—at individual, organizational, and system level—how clinical and nonclinical staff adapted their practices and systems to accommodate and optimize the use of remote consultations both before and in response to the pandemic, and to inform policy going forward. As noted above, our *research* aim was to draw generalizable learning from an in-depth analysis of this case.

The initial evaluation contract was awarded in July 2019 by competitive tender following a public call for proposals. A follow-up contract was awarded in June 2020 using COVID-19 emergency procurement regulations. Ethics approval was obtained from London – Camberwell St Giles Research Ethics Committee (ref 19/LO/0550) and the NHS Research Scotland Permissions Coordinating Centre. A small advisory group was set up within the Scottish government to oversee the project. Progress of the second phase of the evaluation was also monitored by an external advisory group with wide stakeholder representation and a lay chair established to oversee a number of rapid-response research studies on remote care occurring during the pandemic. Fieldwork was conducted by JW and TG.

### Research Questions

Our research questions were, in relation to this national case study:

- What were the multiple interacting influences (clinical, social, technical, organizational, regulatory, and so on) on the uptake, implementation, scale-up, acceptability, effectiveness, and appropriateness of video consultations?
- What was the impact of the COVID-19 pandemic on the scale-up effort?
- What can we learn from this case about the kind of knowledge, capabilities, and infrastructures needed to support the introduction and use of video consultations in different parts of a public-sector health service?

### Study Design

This was a mixed methods naturalistic case study, using an “n of 1” hermeneutic approach drawing on the theoretical work of Flyvbjerg [47], Stake [48], Tsoukas [49], and Cooperrider et al [39]. These authors emphasize the use of narrative methods and rich description to produce a unique account of the case for its own sake. They warn against imposing a rigid analytic framework, producing abstracted models, or getting drawn into disjunctive theorizing (dividing the data into formal themes and categories which are then separately theorized, resulting in a neat but reductive account) [50]. Rather, as Tsoukas explains, the free-text narrative form is used to produce *conjunctive* theorizing—that is, producing an account which weaves multiple themes and influences together in a way that conveys the complexity, historical emergence, and inherent messiness of the case and draws attention to the interdependencies between different aspects of it. Appreciative inquiry applies this methodology to largely successful cases to gain insights particularly—though not exclusively—from what went well [39].

### Theoretical Framework

**Figure 1:**
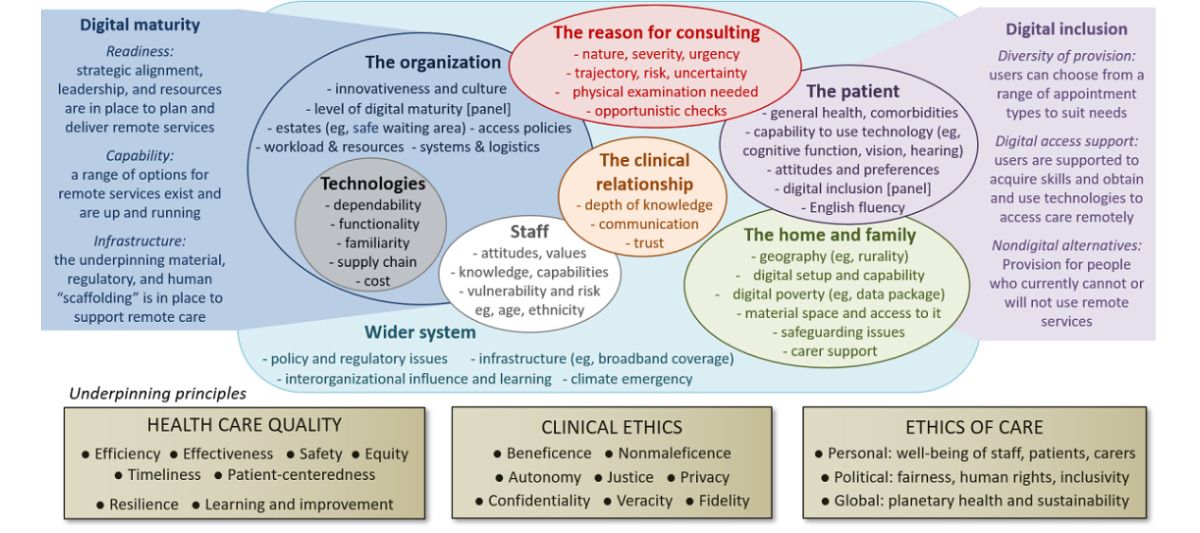
Planning and Evaluating Remote Consultation Services (PERCS) framework.

Development and refinement of the PERCS framework (Figure 1) are described in detail elsewhere [51]. PERCS—which is specific to remote consultations—is an adaptation of a previous framework (nonadoption and abandonment by individuals, and challenges to scale-up, spread and sustainability [NASSS]) of technological innovation in health and care [52], which in turn built earlier work on diffusion of innovations in health care [53]. The domains of the PERCS framework are explained in Multimedia Appendix 1.

### Data Collection Procedure

Data were collected in 2 phases, before and during the COVID-19 pandemic. The periods and data sources for the 2 phases are presented in Table 1.

**Table 1:** Data sources for the 2 phases of the evaluation.

Periods and data sources	Phase 1 (before the pandemic)	Phase 2 (in pandemic)	Total
Period of data collection	July 2019 to February 2020	July to October 2020	12 months
Number of health boards included	8^a^	8^b^	10
Ethnographic observation	60 hours in 11 clinical sites	No ethnography possible due to the pandemic	60 hours
Interviews	140, mostly conducted face to face	83 conducted remotely (mostly by video)	223 (36 were interviewed in both phases)
Interview participant characteristics	Doctors (n=29), nurses (n=18), allied health professionals (n=22), health support workers (n=3), managers (n=18), admin/IT^c^ staff (n=17), patients/carers (n=21); national stakeholders (n=12)	Doctors (n=30), nurses (n=5), allied health professionals (n=13), managers (n=11), admin/IT staff (n=7), national stakeholders (n=17)	Doctors (n=59), nurses (n=23), allied health professionals (n=35), managers (n=29), admin/IT staff (n=24), patients/carers (n=21), national stakeholders (n=29), health support workers (n=3)
Documents	National (eg, on technology-enabled care strategy) and local (eg, protocols)	Relating to pandemic response (eg, remobilization and recovery plans)	N/A^d^
User experience surveys conducted online after consultation	Patients (n=679), staff (n=755)	Patients (n=18,915)	20,349
Patient and public engagement survey	N/A	Patients/public (n=4197), staff (n=1203)	5400
Uptake statistics for the Near Me service, by health board and clinical specialty	January to December 2019	January to September 2020	21 months

^a^Health Boards included Forth Valley, Highland, Golden Jubilee, Grampian, Greater Glasgow and Clyde, Lothian, Orkney, Western Isles.

^b^Health Boards included Grampian, Greater Glasgow and Clyde, Dumfries and Galloway, Highland, Forth Valley, Fife, Orkney, Western Isles.

^c^IT: information technology.

^d^N/A: not applicable.

In sum, our data set comprised 223 interviews with patients, staff, technology providers, and policymakers (further details on participant characteristics are provided in Multimedia Appendix 2); 60 hours of ethnographic observation (including visits to remote settings); local and national documents; and process data such as uptake statistics, patient and staff satisfaction surveys; and patient enablement scores. Participants for interviews were identified in 1 of 3 ways: direct contact (eg, clinicians or managers recommended to us by the Scottish Government or who were listed as having a strategic role); indirect contact (“snowballing” from interviewees by asking them to recommend someone else); and social media (via a Twitter call).

Fieldwork before the pandemic occurred in person but during the pandemic was of necessity conducted remotely. To aid conjunctive theorizing, interviews were conversational in style and lasted between 15 and 60 minutes. Local and national stakeholders were invited to speak about their efforts to develop and scale-up the service. Patients and health and care staff were asked to talk about their experience of video consulting (or why they had chosen not to use this medium). When interviewees talked in the abstract about problems and challenges, we asked them to describe specific examples of these. Qualitative data collection was conducted within a subsample of health boards purposefully selected to explore variation in geography (urban, rural, islands) and progress in the implementation and uptake of video consultations before and during the pandemic.

In addition to qualitative interviews and fieldwork, analysis was informed by evaluation data captured nationally by the TEC team in both phases of the study. A short online survey was completed by patients and clinicians immediately after each video consultation. In phase 1, the patient survey questions focused on experience and perception of technology (eg, usability, call quality). In phase 2, we added a 6-question validated patient enablement instrument to assess perceived quality and usefulness of the clinical aspects of the consultation [54]. The online staff surveys captured their experience of, and perceived satisfaction with, the consultation. In total, there were 19,594 patient responses and 755 clinician responses to the surveys.

A public engagement exercise conducted by the TEC team during the pandemic explored perspectives on the mainstreamed use of video consultations during and beyond the pandemic. This included online and paper questionnaires with members of the public (n=4197) and care professionals (n=1203), which were disseminated through a range of national and community networks.

Data on the uptake and use of the Near Me service were captured nationally during Phase 1 and Phase 2, spanning 12 months (January-December 2019) and 9 months (January-September 2020), respectively. The activity data were captured through the Attend Anywhere platform, as opposed to NHS systems, so it was not possible to establish the proportion of video in relation to other appointment types (ie, face-to-face and telephone appointments).

### Data Management and Analysis

Data were pseudonymized by giving each participant a different name. A spreadsheet containing real names and pseudonyms was stored securely in accordance with General Data Protection Regulations (GDPR). Interviews were anonymized and stored securely and selective sections transcribed. Interviews were not fully transcribed, partly for resource reasons and partly to avoid loss of overview, because salient issues were often captured succinctly in field notes. We returned to the audiotape to obtain a verbatim record where needed. We organized and gained initial familiarity with our qualitative data by organizing field notes and interview notes into an Excel spreadsheet to identify emerging themes. Each row represented an interviewee and each column represented a thematic category. We then considered each thematic category in turn, along with interactions and interdependencies. Following this familiarization phase, we undertook a more theoretically driven analysis using the PERCS framework described above so as to highlight how multiple influences interacted dynamically and unfolded over time.

Quantitative data were used to illustrate and affirm the narrative and inform ongoing data collection and analysis. Uptake and use across different health boards and specialties (and how these changed over time) were used to provide a national picture on the pace and scale of rollout, and highlight areas of focus for ongoing fieldwork and interviews (eg, to explore difference in use across settings and specialties). Survey and questionnaire data were analyzed using descriptive statistics in order to provide a national-level account and explore perspectives across different specialties and regions.

## Results

### Overview

Phase 1 (before the pandemic) of the evaluation generated over 300 pages of interview transcript, field notes, and document excerpts as well as raw quantitative data on staff and patient experience. At the time, our analysis (described in the official report [55]) focused mainly on the question of effectiveness, cost-effectiveness, and sustainability of the service in the context of Scotland’s general policy priorities. Our formal evaluation report of the pandemic period is also available online [56].

In the prepandemic phase, we were particularly struck by 3 things. The first was the focus on region-by-region quality improvement. As described above, there had been long-established strategic drivers for Near Me in Scotland, with strong national policy support for such systems to reduce the human, financial, and environmental burden of travel. Commencing 2017, in one of Scotland’s largest geographic regions (Highland), systematic efforts had been made to work collaboratively with clinicians and patients to implement a video consultation service. By 2019, this work was well underway in Highland, led not by a technical expert but by a clinician (pharmacist) who was well-regarded regionally and had previously been trained in—and personally inspired by—the system-wide approach to quality improvement promoted by the US Institute of Medicine [57]. Building on these developments, work commenced in one other health board (Grampian) to mainstream Near Me, and small-scale implementation began in the other health boards across Scotland. Thus, Near Me had, from the outset, an “organic” (locally grown and locally owned) ethos and a sense that it was being developed to improve access, reduce inequalities, and help save the planet.

The second striking feature of the service in late 2019 was the emergence of different service models reflecting Scotland’s remote geography. At that time video consultations were rarely used in primary care, as almost every citizen lived fairly close to a local general practice and doctors in remote areas were generally happy to do home visits to those unable to travel to surgery. Video was largely a secondary and tertiary care service taken up in particular by remote regions. We identified 3 different models of use:

- Hub-home, in which the clinician connects from the clinic (hub) to the patient at home (or some other location via a personal device);
- Dyadic hub-spoke, in which the clinician in a specialist clinic (hub) connects to the patient in a remote health or care site (“spoke”—typically, an unstaffed kiosk equipped with a self-service video screen and connection);
- Triadic hub-spoke, in which the clinician in a specialist clinic connects to the patient in a remote health or care site with an additional staff member present.

Contemporary images of video consultations generally depict some variant of the hub-home model (eg, doctor in clinic connecting directly to patient at home). This was extremely rare in our data set. Most video consultations in the prepandemic phase were organizationally far more complex—involving a triadic hub-spoke model in which a specialist in a secondary or tertiary care center connected with a remote hospital, primary care clinic, or care home and the patient received both technical and clinical support from a staff member such as a nurse, general practitioner (GP), or health care support worker. As we describe below, this unusual and resource-intensive arrangement produced challenges both at the time and—even more so—once pandemic-related infection control measures were imposed.

The third striking aspect of the Scottish Near Me service was its asymmetric development, driven in some places by particular local enthusiasts and thwarted in other places by lack of them. Often, a video service had been established serendipitously—for example, as a specialist consultant who moved away sought to keep some clinical contact with their patients—and maintained through strong working relationships between key members of staff. This patchwork nature of video consultation services had advantages and disadvantages. On the one hand, it reflected the Scottish Government’s enabling (rather than command and control) approach, in which professionals could be creative and locally adaptive. On the other hand, the lack of a centrally mandated policy meant that despite Scotland’s relative success, *most* services still offered few video appointments and many offered none at all.

**Figure 2:**
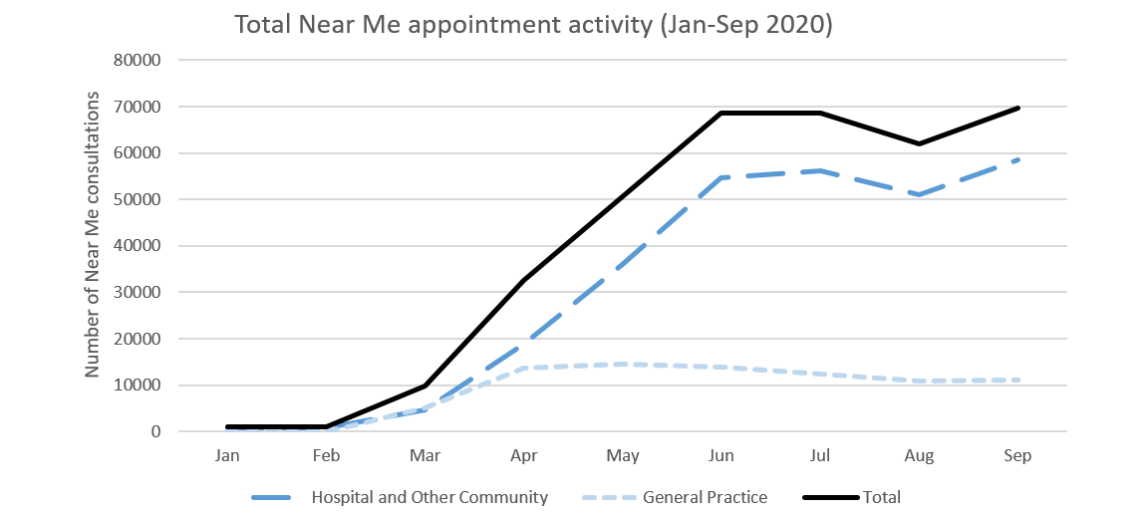
Growth of video consultations before and during the pandemic. The graph shows the total number of video consultations for general practice, hospital, and other community services.

When we returned—using virtual methodologies—to explore the response to the pandemic, our data confirmed a rapid expansion of the service (Figure 2). In 2019, just under 7000 Near Me consultations had been conducted nationally (134 per week on average). In the months preceding the rapid scale-up (January-February 2020), there were approximately 230 video consultations per week. Between March and June 2020, the number of video appointments increased 50-fold, from about 330 to 17,000 appointments per week nationally, and over 50 clinical specialties introduced video consultations for the first time. Other forms of remote consulting were used (eg, telephone and emailing or uploading of photographs). Unfortunately, relative proportions of these different modes could not be accurately captured for further analysis.

Although the pandemic saw a significant shift in the use of Near Me at a national level, the extent of this change varied across care settings. For instance, while many general practices introduced the Near Me service model, most used it infrequently and ad hoc, so that general practice as a whole accounted for only 22.55% (81,822/362,828) of all video consultations in Scotland. Among hospital and community specialties, the services accounting for most video activity were psychiatry, psychology and community mental health (36.41% of all hospital and community care activity, 94,876/260,547), physiotherapy (8.79%, 22,909/260,547), and pediatrics (7.81%, 20,354/260,547).

In the analysis below, we present findings from each domain of the PERCS framework. Interview quotes are provided by domain in Multimedia Appendix 3.

### The Reason for Consulting

Prior to the pandemic, almost all new consultations in secondary care were face to face, in order to establish a clinical relationship, undertake a full physical examination, and conduct baseline assessments (eg, a standardized severity score for rheumatoid arthritis). Video consultations were used primarily for routine follow-up of chronic, stable conditions, especially to convey test results and affirm that the patient remained asymptomatic (see quote Q1a in Multimedia Appendix 3).

Other services with high use of video consultations before the pandemic included physiotherapy, speech and language therapy, pediatrics (for nonacute conditions such as gait abnormality), community mental health follow-up (eg, for patients with depression), and postoperative follow-up. In many such situations, clinicians saw value in “eyeballing” the patient (ie, a visual overview, albeit via video) to assess their general health. Some remote physical examinations were possible, especially by experienced practitioners (Q1b in Multimedia Appendix 3).

During the pandemic, use of video was extended to embrace a much wider range of clinical conditions and reasons for consulting. This was driven by a concerted national effort to maintain the provision of priority clinical specialties (eg, oncology, maternity, mental health), as well as local teams seeking to maintain some service provision across a range of different specialties. Most of these required no formal physical examination. Video was noted to be very useful in psychiatry, psychology, counseling, respiratory medicine, and speech and language therapy, where visual, nonverbal communication was important and personal protective equipment might interfere (Q1c in Multimedia Appendix 3). Some clinicians talked of the need to *feel* a lesion as well as see it (eg, palpating regional lymph nodes in cases of possible cancer), which precluded video examination.

Some conditions for which a visual examination was crucial were considered unsuitable for video consultations. For skin lesions, for example, the variable quality of the video image transmitted from a patient’s device was not always adequate to make a confident diagnosis; instead, patients were often encouraged to obtain and upload a high-quality still photograph taken in good light. This approach also allowed the image to be stored and, where necessary, sent on for a specialist opinion. Most acute ophthalmology consultations required slit-lamp examination or fundoscopy (use of high-intensity light and magnification to see inside the eye) even when there was an evident lesion on the eye (and especially when there was not). While video was used for remote examinations by ophthalmologists, patients still needed to be physically co-present with the optometrist in order to capture magnified images of the eye through specialist equipment. In sexual health, use of video and still images was limited by legal and regulatory restrictions on transmission of intimate images (and by practitioners’ discomfort about exchanging such images).

Both before and during the pandemic, video consultations were widely viewed as clinically less appropriate for poorly defined and less predictable conditions, rare conditions (ie, those with which the clinician and system were unfamiliar), life-changing diagnoses, planning of major interventions (eg, bone marrow transplant), unstable or unpredictable psychiatric conditions (eg, severe emotional trauma, psychosis), or when the patient would need to attend in person anyway to have tests or collect medication. The threshold for offering such patients a remote consultation changed, however, as the risk–benefit balance was dramatically altered by the pandemic. In the context of pandemic restrictions, GPs considered telephone adequate for most problems in known patients, as they felt they rarely needed to rely on visual assessment or physical examination. But video was sometimes considered crucial for visual assessment to exclude rare but potentially serious acute problems such as infection (Q1d in Multimedia Appendix 3).

Primary care is traditionally the “risk sink” of a health service: most new problems are low risk and self-limiting, and potentially serious symptoms or signs can be observed and referred on as needed. During the pandemic, GPs talked of the difficulty in managing risk without the option of bringing the patient in for a face-to-face examination, especially because secondary care colleagues did not always trust an assessment made by telephone. One experienced GP felt they had picked up a possible malignancy purely from the history, so referred the patient on the basis of that, but the patient was “triaged” at the secondary care end and the appointment refused (Q1e in Multimedia Appendix 3).

### The Patient

Our prepandemic evaluation identified numerous comorbidities and preexisting conditions which affected patients’ ability to use the video technology. These included temporary or permanent confusion or other cognitive limitation, visual impairment, or serious anxieties about the technology (including body image concerns about being seen, or seeing themselves, on video display). But more commonly, the barrier to using video was the patient’s general level of debility (Q2a in Multimedia Appendix 3).

Many patients with deafness and partial hearing loss found remote consultations by telephone impossible, while those able to lip-read or use the chat function often welcomed the option of video. Professional interpreters using the British Sign Language were available for some via remote triadic consultations, depending on the availability of interpreters and organizational structures in place to coordinate this. During the pandemic, patients with hearing impairment—and staff—were greatly handicapped by the requirement to use face coverings in face-to-face settings, making a video consultation a more attractive option (Q2b in Multimedia Appendix 3).

Interpreting services for patients who spoke limited English were rarely available by video before the pandemic, due to logistical challenges of bringing the interpreter into a 3-way call. However, remote interpreting services gained impetus during the pandemic, when resources were directed into meeting the technical and logistical challenges (Multimedia Appendix 3, Q2c and 2d).

Before and during the pandemic, lack of familiarity and low digital literacy explained some patients’ reluctance to use video even in the absence of a relevant disability or co-existing condition. While the pandemic provided impetus for upskilling, it was difficult for clinicians or support staff to estimate a patient’s likely capability prior to the consultation, and making decisions based on age, gender, or social stereotypes felt unprofessional (Q2e in Multimedia Appendix 3). Older patients and those assumed to be unfamiliar with digital technology (eg, those in manual and outdoor occupations) were often offered telephone rather than video.

For patients able to use video technologies, their attitudes and preferences toward video, telephone, and face-to-face consultations before the pandemic typically centered on the trade-offs between home and work commitments, travel and transport access, the nature of the clinical problem, a desire (or not) to establish or strengthen a personal relationship with the clinician, and sheer convenience (Q2f and 2g in Multimedia Appendix 3).

More prosaically, patients were not always aware of the video option (some informants commented that this needed flagging when the appointment is booked).

During the pandemic, concerns about infection risk became over-riding for most, leading many people to try video consulting for the first time. Findings from the public engagement survey indicated a high level of support for continuing remote consulting beyond the pandemic, partly because people were now familiar with this medium and partly due to on-going anxieties about risk of infection.

### The Home and Family

Our prepandemic visits to Scotland highlighted the very remote settings in which some people lived. Transport links were sometimes few and unreliable (eg, ferry and plane cancellations due to the weather), and staff as well as patients spent many hours (and sometimes whole days) traveling to and from clinics. Even before the pandemic, many people described how video consultations had transformed their lives simply by saving travel time. But connecting from home was far from an instant option for some patients. Many homes in remote areas were small, of basic construction, and had limited privacy. In some communities (especially certain inner-city areas), there were high levels of poverty and deprivation. In the public engagement survey, respondents’ reported “very significant” barriers to uptake including lack of access to an appropriate device (23.04%, 924/4010), poor internet connectivity (29.92%, 1200/4011), restrictions on mobile data packages (17.07%, 685/4012), and lack of private space at home (19.61%, 786/4009).

Requirements for physical distancing and managing risk of infection during the pandemic required a shift from the triadic hub-spoke model of video consulting to a hub-home model, for which neither the patients nor the service were fully prepared. Failed attempts at video consultations to patients affected by digital poverty (eg, no smartphone, no webcam, limited data package) were common and frustrating (Q3a in Multimedia Appendix 3). Video consulting to home could also mean an inadequate material space (not everyone had a desk or table for example; some consulted from their cars, their bed, or even the bathroom) and potential distractions (especially from children either present in the room or unsupervised somewhere off camera). Some interviewees expressed concerns about the possibility of a patient’s abusive partner listening in.

Patients with low digital literacy or confidence sometimes benefited from on-hand carer support for video consultations; those lacking such support were often limited to telephone. Carer support included setting up and troubleshooting the video link, adjusting the camera angle to facilitate a remote physical examination, preparing and overseeing a child’s appointment (while enabling rather than interfering with the direct clinician–child interaction), and assisting with translation or communication (Q3b in Multimedia Appendix 3).

As we have shown previously, supporting a relative’s remote clinical examination can be emotionally as well as technically challenging, because it may involve complex negotiations between carer and patient about the balance between assistance and autonomy [58]. During the pandemic, shielding and physical isolation measures limited the availability of carer support. The quality of video consultations with care home residents also depended on care workers’ varying technical knowledge and skill.

### The Clinical Relationship

An established clinical relationship, based on previous face-to-face encounters, made clinicians and patients more relaxed about video consultations and allowed clinicians to tolerate the higher levels of uncertainty associated with this medium. Prior to the pandemic, most clinicians liked to have an initial face-to-face consultation to establish rapport and confirm suitability of video for follow-up appointments. Initially, the default in all services was for first assessments to be done in person, but the pandemic required many new referrals to be assessed via video. While these posed challenges (chiefly around technical connectivity), many informants told stories of how such interactions had gone surprisingly well.

Mental health specialties in particular considered video important for communicating (Q3c in Multimedia Appendix 3). A recurring theme in our data was the importance of a high-quality technical connection for establishing and building deep therapeutic rapport.

Perceptions on how remote consulting altered the relationship and interaction between patients and clinicians were nuanced and highly contingent upon clinicians’ interaction styles, perceived value of tactile information and facial expression, and the clinical context of the encounter. Some emphasized the therapeutic value of the in-person physical examination and regretted the loss of such contact during the pandemic (Q3d in Multimedia Appendix 3). Others saw video as a way of circumventing the interpersonal barriers created by facemasks (Q3e in Multimedia Appendix 3).

Video was occasionally advocated for more paternalistic reasons. One medical consultant, for example, considered that teenagers did not take telephone consultations seriously and were likely to “pay attention” more if they were seen by video.

### The Technology

Attend Anywhere, the technology used for Near Me across Scotland, is an internet browser–based video technology that can be accessed by a staff member on a work computer or a member of the public using their own device. One defining feature is its “inbound” workflow, which seeks to emulate how patients physically attend their appointments. For example, a single button on a website (or consistent weblink address on an appointment letter) offers a one-stop “virtual front door” for patients. On clicking that link, the patient enters a “virtual waiting room” (potentially managed by a live receptionist), before being invited into the clinician’s virtual consulting room. Because Attend Anywhere does not require the downloading of software or creation of user accounts, it is easier for patients to use securely. The system has also been designed specifically for health and care, with a strong information governance model that was reviewed and endorsed nationally. This helped avoid information governance restrictions which prevail in many health care organizations.

In our prepandemic evaluation, the Attend Anywhere technology was generally considered by staff and patients to be dependable and to produce high-quality video and audio. In most cases (503/662, 75.98%), patients reported no technical problems during their postconsultation survey. Of those reporting technical problems, the issues mainly related to internet connection and audio–video quality (eg, moments of sound loss, lack of synchronization between video and audio), as opposed to complete technical failure or usability issues. Staff and patients told us that ease of use was partly due to the well-designed software, also partly because many services had invested in high-quality peripherals such as screens and noise-cancelling microphones. Additional data from postconsultation surveys are provided in Multimedia Appendix 4.

During the pandemic, 2 problems occurred. First, the shift to hub-home care models (see above) meant that the connection came to depend heavily on patient connectivity and device (and sometimes also on the home connections of homeworking clinicians), leading to loss of video or audio connection or awkward lag (Q4a in Multimedia Appendix 3). Second, the software platform initially came under significant strain due to an unprecedented increase in volume of consultations, resulting in periods of poor service reliability. These problems were dealt with promptly by the technology supplier by removing bottlenecks from the underlying application and increasing server capacity.

The video connection also depended on other technical systems, particularly for accessing the virtual waiting area. For example, text-messaging systems through the electronic booking systems to provide the patient with the URL were prone to error during rapid rollout, sometimes sending incorrect links to patients.

When the video connection failed, telephone was used—fairly unproblematically—as a backup.

Despite these challenges, patient survey responses remained generally positive. Most (14,677/18,817, 78%) reported no technical problems. Of the remainder, most problems were similar to those encountered in the prepandemic evaluation (eg, audio or video quality).

The functionality of Attend Anywhere was considered good by most interviewees. Clinicians particularly liked the virtual waiting room and the option for screensharing (Q4b in Multimedia Appendix 3). This functionality, however, required a reasonable screen size and was of limited use if the patient was using a smartphone or small tablet device.

While Attend Anywhere was unfamiliar to most patients, the “inbound” workflow with a single point of entry and virtual waiting area made sense to patients because (they told us) using the technology “felt like” going into a clinic and physically sitting in a waiting room—a finding that others have also observed [59]. There were occasional glitches such as when a patient, offered several waiting rooms, selected the wrong one (Q4c in Multimedia Appendix 3). In the larger services, an actual live receptionist would meet and greet the patient on the video call and transfer the patient to the correct virtual waiting room.

A national policy decision to provide Attend Anywhere to all NHS organizations, alongside financial investment in the model in 2018, strengthened the organizational incentives to expand use of the technology prior to the pandemic. At the start of the pandemic, central procurement of Attend Anywhere was further extended by 2 years. Significant challenges were faced in resourcing laptops, video cameras, and audio equipment in the face of national shortages and disrupted supply chains during the pandemic. But because Attend Anywhere is an encrypted browser-based technology, some staff could make use of personal devices to run video consultations.

### Staff

Staff attitudes toward video consulting varied considerably, especially before the pandemic. Most clinicians we spoke to who had used Near Me had positive things to say about it, describing it as a significant way of improving patient access and experience by reducing the need for travel, providing faster and more direct access to specialists, and helping overcome reluctance to visit clinical spaces. Some clinicians, who used video never or rarely before the pandemic, depicted such services as unprofessional or unsafe (Q5a in Multimedia Appendix 3). But interviews during the pandemic found that many had changed their perspective (Q5b in Multimedia Appendix 3).

Many clinicians talked about still being on a learning curve about when to offer the remote option and how to conduct such consultations effectively, including adapting ways of interacting with patients to take account of the physical and symbolic differences of the virtual environment (Q5c in Multimedia Appendix 3).

The shift to video was not universally welcomed. Some staff felt its continued use beyond the pandemic was a retrograde step because it was less professionally fulfilling (Q5d in Multimedia Appendix 3).

Before the pandemic, the use of video consulting was almost never spoken of in relation to staff well-being (with the exception of saving travel time). During the pandemic, video consultations were seen, on the one hand, as protecting staff—especially vulnerable ones—from risk of infection. On the other hand, they were described as more cognitively demanding and tiring than face-to-face ones. In a few cases, the clinician described becoming unwell during a video consultation (Q5e in Multimedia Appendix 3).

### The Organization

The general innovativeness and digital maturity of health care organizations had a strong bearing on their ability to introduce, routinize, expand, and evaluate their video consultation service. In this regard, the nationwide effort by the Scottish Government to strengthen digital infrastructure over the previous 10 years was evident. Many, though not all, organizations had good broadband connection, adequate hardware, and sufficient numbers of trained staff to implement the technology. In many specialties, the equipment had been installed but had not been routinely used until pandemic pressures created an impetus.

Developments before the pandemic revealed the importance of equipment setup and availability. This included dual screens, high-quality cameras and noise-cancelling microphones and speakers, as well as specialist equipment (eg, high-quality audio headsets for speech and language therapists, high-magnification cameras for dermatologists). Much work had gone into these details alongside the rollout of Near Me in 2018-19. However, during the early stages of the pandemic, demand for this kit soon exceeded supply, especially because infection control protocols prevented sharing between staff. Some ran short and had to rely on the phone.

Video consulting relied on other technical systems, such as electronic booking and secure asynchronous communication channels with patients (eg, texting, email). Digital maturity, in this regard, was the extent to which standardized processes had been established for the smooth running and reconfiguration of appointment schedules to accommodate different modalities. The rechanneling of IT and outpatient resources during the pandemic helped address these challenges, but staff in some settings described various glitches, such as patients entering incorrect virtual waiting areas, due to rapid restructuring of administrative workflows and systems (Q6a in Multimedia Appendix 3).

Prepandemic infrastructure strengthening through the TEC Programme had mainly focused on outpatient hospital sites, which were able to scale-up quickly as the pandemic hit. By contrast, general practice services had had little interest in video consultations before the pandemic and had to be rapidly set up in early 2020. Because of shielding, and also because clinic space was repurposed (eg, for seeing potentially infected patients), many general practice staff worked from home. Despite input from mobile IT teams to install the necessary equipment, this remote working did not always go smoothly (Q6b in Multimedia Appendix 3).

A major challenge across all sites was establishing adequate space and equipment for a video consultation. During the pandemic, staff worked pragmatically and adaptively, with technology to hand. Particularly for the larger city hospitals, moves toward hot-desking and shared office space were not conducive to the expansion of video services, because open-plan working encroaches on privacy and may require the clinician to wear a mask.

Some staff described logistical barriers to establishing and running a remote consultation, notably requesting and obtaining blood test results from the patient’s local primary care practice, transmitting a prescription to the patient’s local pharmacy when the patient was not there to collect it in person, providing patients with printed information sheets, or obtaining written consent (Q6c in Multimedia Appendix 3).

Many of these logistical issues required a redistribution of resources across the system (eg, additional staff were needed to run a more complex appointment system and virtual waiting area). The expansion of video appointments also required new ways of working and sharing data across departments and with patients (eg, respiratory services purchased pulse oximeters for patients to use at home). While these changes were initially developed (and resourced) as an interim “workaround” measure, there is both enthusiasm and concern for sustainability of these practices.

Before the pandemic, efforts to introduce and use remote services often stalled because of staff shortages (especially when senior clinicians were replaced by a series of short-term locums) or general lack of resources. Attempts by IT and service managers to set up remote services and embed them in business-as-usual were dependent on clinicians who were willing to join the change effort, use the technology, and consider working in a different way. Such individuals were relatively rare (described by one interviewee as the “keenies” but perhaps more formally classified as “innovators” or “early adopters” [53]). The rechanneling of local resources during the pandemic, alongside a lull in routine activity in some specialties, provided clinicians with the opportunity to try out and adapt new ways of working.

We found in our prepandemic evaluation that both dyadic and (even more so) triadic hub-spoke models raised logistical challenges and required various kinds of double-handling (eg, appointments needed to be made, rooms booked, and staff members made available, at both the hub and the spoke site). In some remote sites, there was much redundancy (eg, staff were allocated to a hub clinic for a whole morning but only 1 or 2 patients were seen); in others, a lone staff member had to juggle multiple roles (Q6d in Multimedia Appendix 3).

Because much of this double-handled activity related to outpatient consultations or cold surgery, it was stalled during the pandemic. It is unclear how, as video consultation services expand beyond the pandemic, this issue will be resolved.

All the organizations we studied were committed to a policy of inclusion. Service teams were encouraged to ensure that new remote models did not disadvantage people in relation to service access and allowed patients to exercise choice where clinically appropriate.

During the pandemic, patient choice was heavily constrained by infection control protocols, a measure that created huge challenges for ensuring equity of access. Some of our respondents were keen that video should not be the default option for everyone going forward (Q6e in Multimedia Appendix 3).

### The Wider System

The Scottish Government’s longstanding commitment to using technologies to achieve high-quality, accessible, and equitable care and contributing to a low-carbon future created an important national-level context for the introduction and mainstreaming of video consultations. While remote areas had limited or no broadband access, this was improving as a result of a policy push for connectivity. However, there was no strong tradition of digital communication (some outlying islands, for example, had only had broadband outside the largest town for a few years, so not everyone owned, or was comfortable using, a smartphone). When the pandemic hit, the Near Me service was immediately mandated across the country, allowing rapid and consistent implementation locally and regionally (Q7a in Multimedia Appendix 3).

Also important was engagement of professional bodies such as Royal Colleges, who endorsed the TEC Programme’s vision and guidance documents from an early stage. Proactive communication between government and professional bodies ensured that frontline clinicians believed that the changes were professionally endorsed and led rather than imposed by central government. In addition, the TEC team worked with NHS National Services Scotland (a public body that provides national strategic support to NHS services) to review and monitor network bandwidth capacity for the rapid expansion of video, and sought data protection approvals at a national level to provide confidence and continuity across local organizations.

There was a concerted effort at national and local level to support collaborative learning and interorganizational support. This included shared learning within specialties at national level (supported by NHS Education Scotland) and board-level collaboration between departments about local processes (Q7b in Multimedia Appendix 3). As well as supporting knowledge sharing across the 14 health boards, the Scottish TEC team engaged with national and regional leads across England and Wales to facilitate local rollout shortly prior, and during, the pandemic. This included the sharing of training resources, patient facing materials, and governance documentation. The English and Welsh NHS Trusts were also hosted temporarily on the Scottish Attend Anywhere platform to help them off the ground until separate platforms could be established, at some risk to their own system integrity.

## Discussion

### Principal Findings

This is a mixed methods case study of the development and pandemic-driven scale-up of video consultation services across Scotland. Using the PERCS framework, we have mapped a complex data set of qualitative and quantitative findings to explain multiple interacting domains of influence. Before the pandemic, a national program to extend a service that had been successful in local pilots was already underway, driven by an ethos of collaborative quality improvement, reducing inequalities, and achieving cross-government low-carbon goals. By the time the pandemic hit, there had been considerable investment in material and technological infrastructure, staff training, and professional and public engagement. Scotland was thus uniquely well placed to expand its video consultation services at pace and scale, resulting in a dramatic increase in number of services using video. While not everything went smoothly, video consultations became available as business-as-usual for a much wider range of clinical problems, vastly extending the prepandemic focus on outpatient monitoring of chronic stable conditions.

### Strengths and Limitations

The great strength of this study is that, somewhat serendipitously, we had built a good working relationship with the Scottish Government and many regional implementation teams just before the pandemic hit, and were able to mobilize quickly to undertake a second phase of the evaluation. This meant that—perhaps uniquely in any country—we obtained both pre- and peripandemic data of various kinds. We were also undertaking other research on remote consultations across the UK and developing the PERCS framework, which proved useful for explaining and organizing multiple streams of data.

The limitations of this study are threefold. First, pandemic restrictions meant that we could undertake no ethnographic work in phase 2, and our data collection more generally was affected by the unprecedented pressures on NHS staff (who, for example, had had little time to reflect individually or collectively on what was happening). This also raises potential sample biases toward more technically literate participants (ie, to conduct interviews by video), as well as those individuals with the time available to speak with us. We sought to mitigate these issues by offering phone as well as video interviews and adapting interview schedules to meet individual circumstances. Second, our positive and now longstanding working relationship with national- and regional-level stakeholders may have led us to view their change efforts in a positive light, though in other large-scale evaluations we have had equally positive relations with stakeholders but produced less positive reports [21]. Third, the pace, scale, and scope of the evaluation did not allow us to produce an economic component to explore the costs and cost-effectiveness of the video option in different circumstances and settings.

### Comparison With Prior Work

Scotland’s story of scaling up video consultations resonates with what we know of other countries that had a relatively advanced infrastructure for telehealth—for example, Australia, where a small (and possibly skewed) survey found that up to 60% of health professional respondents had consulted by video during the pandemic, aided by a slackening of regulatory restrictions and more flexible reimbursement [35]. In Norway, the relative proportion of remote GP appointments increased from approximately 3% (before the pandemic) to almost 60% during the initial lockdown [60]. This move was encouraged by the Norwegian Ministry of Health and Care services and incentivized by temporary modifications to reimbursement systems. Clinician surveys revealed that, while such shift raised new possibilities for video consulting in the longer term, important clinical, technical, and operational challenges remain [60]. In New Zealand, the Royal College of General Practitioners urged all members to switch to remote (video, phone, email) consultations with a goal of reducing in-person visits by 70% within 48 hours of the national lockdown [61]. This rapid response was aided by a NZ $20 (US $13.8) million government commitment to increase telehealth capacity, training webinars through the National Telehealth Resource Center, and temporary relaxation of electronic prescribing rules [62]. While nationwide uptake of this service model is yet to be comprehensively reviewed [63], preliminary research indicates longstanding potential; pending further investments; and closer attention to IT infrastructure, regulatory, and accessibility considerations [64,65]. We suspect there will be useful comparisons between New Zealand and Scotland, possibly using the PERCS framework.

Basu et al [27] have sought to capture the different perspectives of the International Medical Informatics Association Telehealth Working Group, to broadly explore the role of telehealth in 10 different countries during the pandemic. The authors present 6 themes which align broadly with our Scottish case study, namely, strategic (policy decisions and legal changes); operational (increasing capacity and delivery by building skills and resources at pace and scale); regulatory (including pandemic-related unofficial workarounds with unregulated products); changes in attitudes and uptake; public engagement; and training and education. The authors use the World Health Organization Health System model to emphasize the sociotechnical nature of these changes. Other system-focused frameworks such as i-PARIHS (integrated Promoting Action on Research Implementation in Health Services) take a similar though not identical approach [66].

In the language of system innovation, the pandemic was what Van de Ven [67] would call an “environmental shock”—something that generates uncertainty, puts organizations under stress, and requires an urgent adaptive response. A weak system is highly vulnerable to such shocks but a resilient one that is able to adapt can sometimes use the shock as an impetus to innovation and thereby become better able to weather the next shock [68]. The litmus test for Scotland is perhaps not the impressive expansion of video consulting during the pandemic under emergency measures (unregulated telehealth) but the extent to which the positive elements of this expansion will be retained and mainstreamed once such measures are rolled back (regulated telehealth) [27]. It is to Scotland’s credit that both its technological and human infrastructure were sufficiently resilient to respond in ways that could often be routinized within the existing system.

Gkeredakis et al [28] apply 3 perspectives to shed light on the varied uses of digital technology, and associated tensions, during the COVID-19 crisis: *opportunity* for accelerated innovation and removal of barriers to experimentation; *disruption* to organizational and occupational practices, generating new dependencies and risks; and *exposure* of vulnerabilities in both people and infrastructures that have previously gone unnoticed, such as physical work spaces, IT networks, and key workers [28]. Our findings illustrated each of these themes.

### Conclusion

Scotland’s national-level groundwork before the pandemic allowed many services to transform, at pace and scale, to a video-first mode of operating. Key contributors included the “burning platform” of the pandemic, national strategic vision, a well-resourced quality improvement model based around communities of practice and system learning, dependable technology, and multiple opportunities for staff to try out the video option.

We anticipate that sustaining video as the new normal will depend on multiple issues such as digital infrastructure, human and financial resources (distributed fairly across the system), training (including digital literacy and teleconsulting skills), workforce (including extent to which video can help compensate for staff shortages), data security (including overcoming the tendency for regulations to be overly restrictive), and research into remote clinical examinations [25,69].

Evidence that remote care contributes significantly to greener health services is currently limited (one study suggests that a substantial reduction in carbon footprint could be made [70]), but we and others are continuing to study this important factor. Further research is also recommended into different service models. We flagged, for example, that the triadic hub-spoke model is somewhat labor intensive and unlikely to be scalable, whereas hub-home is limited by patients’ digital and material setup. Other models have also been described [71].

Scotland provides an important national case study from which other countries may learn. We invite others to apply the PERCS framework to their own case studies and propose refinements to it.

## Appendix

Multimedia Appendix 1 Planning and Evaluating Remote Consultation Services (PERCS) domain explanations.

Multimedia Appendix 2 Interview participant characteristics.

Multimedia Appendix 3 Qualitative quotes.

Multimedia Appendix 4 Postconsultation survey data.

## Abbreviations

GDPR: General Data Protection Regulations
i-PARIHS: integrated Promoting Action on Research Implementation in Health Services
IT: information technology
NASSS: nonadoption and abandonment by individuals, and challenges to spread, scale-up and sustainability
NHS: National Health Service (UK)
PERCS: Planning and Evaluating Remote Consultation Services
TEC: technology-enabled care
