# Supplementary material for: Expanding Video Consultation Services at Pace and Scale in Scotland During the COVID-19 Pandemic: National Mixed Methods Case Study
Source: J Med Internet Res. 2021 Oct 7;23(10):e31374. doi: 10.2196/31374 (PMC8500351; doi:10.2196/31374)
Supplement: Multimedia Appendix 1 [file jmir_v23i10e31374_app1.docx]

## Multimedia Appendix 1: PERCS domains explained

- *The reason for consulting* considers not just the illness or condition but why the patient wishes to be seen (or why the clinician wishes to see them) now*.* It thus draws attention to the urgency and rate of progression of the presenting problem, whether the appointment is patient- or clinician-initiated, and whether (and what) advice or treatment is being requested.
- *The patient* domain includes attitudes towards illness and remote consulting, which in turn are influenced by their identity, values, personality traits, beliefs, health and digital literacy, and lived experience of illness or disability.
- *The clinical relationship* includes trust and positive regard between parties, often though not always linked to duration of relationship. It also embraces the clinician’s—and in some cases the administrative team’s—knowledge of the patient and their illnesses and consulting patterns.
- *The home and family* includes how the material features, physical layout and symbolic spaces of the home influence issues such as privacy and comfort when consulting remotely [51]. Disadvantaged people may have no home at all, or a home which is small, crowded, lacking privacy or not connected to the Internet. This domain also includes socio-cultural aspects of family life—for example, family structure, English fluency and who controls access to the home computer—as well as the education and digital literacy of family members who could potentially support the patient.
- *Technologies* includes the functionality, technical performance and ease of use of key technologies as well as their dependability and familiarity (the telephone, for example, is dependable and familiar though it lacks the functionality for a visual channel). This domain also incorporates the technology supply chain (including the risks of a sole supplier pulling out) and the costs of both up-front investment and ongoing maintenance and repair, as well as how these costs are distributed across the system. It thus subsumes the ‘value proposition’ domain from the NASSS framework [49].
- *Staff* embraces staff attitudes, which are grounded in professional norms and values including those relating to quality and safety of care, the digital literacy and confidence of staff members, and—in the context of the pandemic—staff vulnerability to infection and levels of exhaustion. Aspects of staff members’ home environment may be relevant if working from home.
- *The healthcare organization* domain includes innovativeness, readiness and normalization efforts. Innovative organizations tend to be large, functionally differentiated, non-hierarchical and with adequate slack (people and resources that can be channelled into new projects to get them up and running) [50]. Readiness for innovation requires both top and middle-management support, absence of opponents, and assessment of innovation-system fit (e.g. a business case), though such requirements were relaxed during the pandemic. Normalization of technologies within organizations includes supporting staff to make sense of the new technology in the context of their work; engaging them to participate; coordinating efforts to implement; and monitoring benefits and costs [52].
- *The wider system* includes aspects of the policy context, including policy attention to such things as planetary health and social inequalities. It also includes infrastructural elements such as broadband availability, and the nature and extent of inter-organizational influence and learning in which early-adopting organizations pass on insights and resources to those coming on stream later [50].

The PERCS framework also includes two side panels—*digital maturity* (of the organization) and *digital inclusion* (for the population it serves). Whilst these concepts are to some extent covered within the central domains, the terms are increasingly widely used in healthcare circles and are worth exploring in more detail.

- A *digitally mature* organization has the readiness, capability and infrastructure to assimilate, test and routinize new technologies into business as usual [53-56].
- *Digital inclusion* embraces three kinds of measures designed to ensure that the less digitally-equipped and digitally-confident are not disadvantaged: diversity of provision, support for digital access, and availability of non-digital alternatives [57-62].

The seven domains listed above interact and evolve dynamically over time, which is why a PERCS analysis is best undertaken longitudinally using narrative as a summarising and synthesising tool.
