# Supplementary material for: Expanding Video Consultation Services at Pace and Scale in Scotland During the COVID-19 Pandemic: National Mixed Methods Case Study
Source: J Med Internet Res. 2021 Oct 7;23(10):e31374. doi: 10.2196/31374 (PMC8500351; doi:10.2196/31374)
Supplement: Multimedia Appendix 2 [file jmir_v23i10e31374_app2.docx]

## Multimedia Appendix 2: Interview participant characteristics

| Stakeholder groups | Interviews |
| --- | --- |
| Doctors | 48 |
| *GPs (19)*  *Consultants (27)*  *Dual accredited (2)* |  |
| Nurses | 21 |
| Allied health professions | 31 |
| *Speech and Language Therapists (8)*  *Physiotherapists (7)*  *Dietitians (4)*  *Counsellors (3)*  *Psychologists (5)*  *Pharmacists (2)*  *Occupational Therapist (2)* |  |
| Health support worker | 2 |
| Third sector support worker | 1 |
| Clinician-managers | 6 |
| Non-clinical managers | 16 |
| Admin / support | 10 |
| EHealth/IT staff | 11 |
| Patients | 16 |
| Relatives/carers | 5 |
| National level stakeholders | 20 |
| **Total** | **187^a^** |

^a^36 participants interview twice (phase 1 and phase 2)
