# Supplementary material for: Expanding Video Consultation Services at Pace and Scale in Scotland During the COVID-19 Pandemic: National Mixed Methods Case Study
Source: J Med Internet Res. 2021 Oct 7;23(10):e31374. doi: 10.2196/31374 (PMC8500351; doi:10.2196/31374)
Supplement: Multimedia Appendix 3 [file jmir_v23i10e31374_app3.docx]

## Multimedia Appendix 3: Qualitative quotes

DOMAIN 1: THE REASON FOR CONSULTING

1. “*New patients we don’t do via video, but for return patients you don’t have to examine.… Say you have someone with lymphoma, and after one or two courses of treatment, there is nothing left to find.*”—consultant haemato-oncologist
2. *“as long as I can see the hand move, I know the tendons are attached”—*physiotherapist describing follow-up after hand surgery
3. “*I could see her, and she could see me, and that made a real difference compared to a telephone call*”—community mental health nurse
4. “*If people are saying they have got a sore knee or sore wrist…. asking them to stand up and show me their knee, and you can immediately see… ok, it is not massively red and not to worry about it…”—*general practitioner
5. *“One patient complained of rectal bleeding. I referred them [without physical examination] as they’d got altered bowel habit. But the surgeons did telephone triage, they said it was constipation, use [symptomatic remedy]. This sort of thing is right at the edge of what we can manage safely remotely.”—*general practitioner

DOMAIN 2: THE PATIENT

1. “*We got him the finger thing [oximeter] but he couldn't really... he couldn't really work it and I just... I felt he was too unwell to allow him... and he was alone. We just needed to see him.”—*respiratory physiotherapist
2. *“Face masks are obviously a massive problem for us. If someone is struggling to hear me, they can look at lip patterns, hands and gestures.”—*audiology consultant
3. “*Trying to get an interpreter three-way conversation is difficult. I have three Polish that have no English at all so that would be face to face…”—* specialist epilepsy nurse, pre-pandemic
4. “*[Pre-pandemic] we would have to bring those people into the clinic. But we couldn’t, so we had to use the three way call for the first time....We would never have thought of that – interpreter was always face to face. But now we don’t need to.”*—service manager, colorectal surgery, during pandemic
5. “*That was probably quite a prejudiced, subjective assessment really. Probably based on age, intelligence level, what their job was.*”—general practitioner
6. *“Patients tend to… want to be seen face-to-face, to be honest… [especially] if it is a new diagnosis or if they’re having major issues”*—consultant geriatrician
7. “*There have been young people who have just preferred the phone, they see it as quicker”*— consultant gastroenterologist

DOMAIN 3: THE HOME AND FAMILY

1. *“I think generally people are struggling with the connection, connectivity – that is the biggest problem. And when it does not work, it really doesn’t work. There is no in-between. It is frustrating for some staff … when expecting to see 5-6 children, and not seeing any of them”—*paediatric physiotherapist
2. *“I send parents a leaflet of what I’m going to do. … The child has to wear shorts, your camera has to be like this etc etc. Important that child is primed that way.”*—paediatric consultant, speaking of orthopaedic assessments
3. “*We do use our nonverbal skills to pick up information—eye contact, facial expressions, state of their house. We all use different things, if they have a sense of humour, how they react to us. So a lot of things, tiny things, that most people don’t use, we do.”—*mental health nurse
4. “*Patients value face to face. Patients value touch. They value touch more than doctors actually, there’s a study on that. Small things like a hand on the shoulder, shaking their hand.*”—general practitioner
5. *“I would rather do the consultation with patient by video so they could actually see my face – rather than a masked person coming towards them… there is no softening of the effect. The face delivers a lot”—*respiratory consultant

DOMAIN 4: THE TECHNOLOGY

1. “*It can be ok as long as good internet connection. But people who …. the picture is fuzzy. The worst thing is the time delay. When you see someone’s lips moving, and then seconds later you hear what they are saying—that is like wading through treacle.”*—psychologist
2. *“So if you are working on a formulation you can share a blank formulation document, you can type into that and the patient can see you typing into that”*—cognitive behavioural therapist
3. “*She wasn’t in the waiting area, so I called her - she’d logged into the Lanarkshire [different region] waiting room”—*psychologist

DOMAIN 5: STAFF

1. *“I can’t stand video consultations. First of all, I’m a doctor. I’m owning my patient. A doctor wants to have direct contact with patients.*”—consultant oncologist
2. *“I was a little sceptical to be honest [before the pandemic]. … Now, I know it works. And I think it is because we have been thrown into the deep end a bit with it.”*—consultant urologist
3. *“I’ve modified my consultation style because of the lag [in sound transmission]. If you don’t do that, there’s a moment of over-talking and interruption. So now I give more time [after speaking] to make sure that the patient knows my speech has been completed. We use silence quite a lot in psychiatry anyway. At the beginning of the consultation I do what I call a ‘technical introduction’—I take 3 or 4 minutes to explain to patient the technological process. There’s a mini-script you need to go to. For example ‘if it cuts out, this is what to do’”*—consultant psychiatrist
4. “*A lot of [my colleagues] would want to go back to face to face. …. Seeing people at home, and we are nice nurses…. People say it is just who we are as nurses. … It is people’s deep held value[s].”—*respiratory nurse who did a lot of home visits
5. “*I said to the patient, ‘I’m really sorry. I’m on the edge of a migraine. I will switch the video off so I don’t feel I have to watch you. You’ll see me looking all over the place, and if you find that weird I can switch my video off.’”*—epilepsy specialist nurse

DOMAIN 6:

1. *“I had one clinic when no one turned up because they were all in the wrong waiting room…I don’t know whether the patient was sent the wrong letter, and put in the wrong waiting room…. It is a whole new learning process for the admin teams.”* – paediatric consultant
2. “*One of my GP partners was shielding for 4 months, they did all their clinical work remotely, but they can be sitting waiting for 90 minutes for it to start working….Trying to log into the practice EPR [Electronic Patient Record] system. Very frustrating.”*—general practitioner
3. *“If they are here [in face to face consultation] and you need to take blood samples, you do it in that room, at that moment. But with these [video] ones there is so much admin—getting them to see their GPs, make an appointment, get a sample, and so on… If they were here, everything would be done in one moment. We often take the blood samples ourselves, and they sign the consent form. So it is little things.”*—genetics counsellor.
4. “*All pre-op patients need height, weight, blood pressure, sats [blood oxygen saturation], and an airway check [the Malampati score], and lots need spiro[metry], ECG, bloods, urine and swabs. The pre-op [specialist] nurses [connecting remotely] have 45-90 minutes for that, but they can’t do any of the extra checks. All that is done by us. They’re in a room up there, we’re in a room down here, we do all the actual things, they just ask the questions. If we do all that work, we need to staff accordingly. Management needs to do the sums. It’s double resourced and double roomed to do via video link.”—*general nurse in remote community hospital
5. *“We need to do more for people who don’t have access to broadband or can’t afford a laptop. … I don’t believe that it [remote] should be the default. We’ve come a long way in healthcare, we don’t want to ruin it now. It really needs to be looked at properly.”*—service manager

DOMAIN 7: THE WIDER SYSTEM

1. “*And when Covid happened – the red tape seemed to vanish, and whether people wanted to do it or not, it just happened very quickly.”*—consultant rheumatologist
2. *“The learning they got from each other. There are national webinars. They have been good. But that local conversation you need… there are very distinct local systems, policies, procedures that you need to work with.”*—project manager
