# Supplementary material for: Expanding Video Consultation Services at Pace and Scale in Scotland During the COVID-19 Pandemic: National Mixed Methods Case Study
Source: J Med Internet Res. 2021 Oct 7;23(10):e31374. doi: 10.2196/31374 (PMC8500351; doi:10.2196/31374)
Supplement: Multimedia Appendix 4 [file jmir_v23i10e31374_app4.docx]

## Multimedia Appendix 4: Post-consultation survey data

**Table S1: Benefits and disadvantages perceived by patients pre-pandemic (N=679)**

| **Benefits** | **% reported** |
| --- | --- |
| Saved travel | 72% |
| Convenience | 62% |
| Saved time | 57% |
| Saved money | 39% |
| Did not have to wait as long for the appointment | 29% |
| Did not have to take time off work | 28% |
| Did not need someone to take me | 23% |
| Because of my condition it was safer/easier | 23% |
| Did not have to arrange child care | 13% |
| Other ^a^ | 2% |
| **Disadvantages** | **% reported** |
| Could not hear the person properly | 7% |
| Could not see person properly | 4% |
| Face to face would have been better | 3% |
| Had to have another appointment face to face anyway | 2% |
| Could not find somewhere private to conduct the call | 1% |
| Took longer to arrange | 1% |
| Use mobile data allowance | 1% |
| Cost money to do | <0% |
| Was too complicated | <0% |
| Other ^b^ | 3% |

^a^ examples of ‘other’ benefits included feeling more comfortable at home and less stress from journey

^b^ the majority of ‘other’ disadvantages related to the technical connection or duration in the virtual waiting area.

**Table S2 Benefits and disadvantages perceived by clinicians pre-pandemic (N=755)**

| **Benefits** | **% reported** |
| --- | --- |
| Saved service user travel | 56% |
| Saved clinician travel | 38% |
| Took less time | 20% |
| Avoided patient travel by plane/ferry | 14% |
| Avoided use of patient transport | 11% |
| Safer/easier because of patient's condition | 10% |
| Other ^a^ | 8% |
| **Disadvantages** | **% reported** |
| The video and audio quality impaired the consultation | 17% |
| Face to face would have been better | 8% |
| Could not do everything via video, needed to arrange another appointment | 2% |
| Service user/patient was uncomfortable with the technology | 2% |
| Appointment took longer | 2% |
| Other ^b^ | 9% |

^a^ example ‘other’ benefits included more rapid assessment of patient, allowing patient to attend while at work and seeing the patient despite adverse weather conditions

^b^ example ‘other’ disadvantages included minor technical issues connecting and assistant needed for the patient using the technology.

**Table S3 Benefits and disadvantages perceived by patients during pandemic (N=18,915)**

| **Benefits** | **% reported** |
| --- | --- |
| Did not have to travel to a consultation | 71% |
| Saved time | 67% |
| Less chance of catching an infection than at a face to face consultation | 54% |
| Did not have to take so much time off work / usual activities to attend | 40% |
| Better for the environment | 34% |
| Improves my access to services | 31% |
| Saved money | 31% |
| Seeing me at home made it easier to explain my situation | 24% |
| Easier to have a relative / carer with me in the consultation | 13% |
| Other ^a^ | 2% |
| **Disadvantages** | **% reported** |
| Could not hear or see the other person properly | 8% |
| We had a poor internet connection | 6% |
| Do not like video calls | 5% |
| Need an extra appointment because we could not complete everything by video | 3% |
| Found it hard to understand the other person by video | 2% |
| Found it hard to make myself understood by video | 3% |
| Could not find somewhere private to make my call | 2% |
| Video call was too complicated | 1% |
| Worried about the cost of mobile data / using my data allowance | 1% |
| Other ^b^ | 6% |

^a^ example ‘other’ benefits included more rapid assessment of patient and allowing access from comfort of home

^b^ example ‘other’ disadvantages included prolonged periods in the virtual waiting area, difficulties dealing with technical delays, challenges/concerns supporting remote examination.
